# Editorial: Sport and community

**DOI:** 10.3389/fspor.2022.1057368

**Published:** 2022-10-13

**Authors:** Kyle A. Rich, Claire Jenkin, Patti Millar, Katie Sveinson, Emma Sherry

**Affiliations:** ^1^Faculty of Applied Health Sciences, Brock University, St. Catharines, ON, Canada; ^2^Institute of Sport, University of Hertfordshire, Hatfield, United Kingdom; ^3^Department of Kinesiology, University of Windsor, Windsor, ON, Canada; ^4^McCormack Department of Sport Management, University of Massachusetts Amherst, Amherst, MA, United States; ^5^Department of Management and Marketing, Swinburne University of Technology, Hawthorn, VIC, Australia

**Keywords:** sport, community, common good, theory, context, culture, interdisciplinary

The sport-community relationship is a tenuous, yet somewhat ubiquitous idea. Sport is often described in academic, policy, and public discourse as an avenue for developing pride (1), civic engagement (2), tourism and economic development (3, 4), social inclusion (5), social capital (6, 7), and a host of other social outcomes. However, these claims are often either tentative, speculative, decontextualized, or poorly understood. Indeed, the advent of sport as a cultural signifier may be a symptom of ever-increasing rhetoric of individualism and market-oriented thinking of neoliberal ideologies and globalization (8)—processes which have undeniably changed public perceptions of sport's relationship with community.

The task of linking sport with any sort of common good is not a simple one. As sport is developed, accessed, and consumed through public, commercial, and civil society organizations, its relationship to community is shaped by varying policies and politics which are developed and enacted differently (9). Sport is therefore understood and enacted within distinct cultural contexts. Further, as sport is also practiced in varying ways (from leisurely, informal engagement to extremely structured and regulated elite competitions), how we define sport in communities, and work to support or develop participation opportunities has also been problematized (10). Therefore, mapping the sport-community relationship is a complex and difficult, conceptual endeavor.

The purpose of this Research Topic was to provide an interdisciplinary and holistic exploration of the sport-community relationship through empirical, theoretical, and methodological contributions. In doing so, we hoped to highlight the various social, cultural, political, and managerial implications of community in and for sport. We aimed to advance the discussion in several ways. Firstly, by engaging perspectives from diverse disciplines such as sociology, anthropology, political science, leisure studies, and sport management, we sought out diverse theoretical and methodological approaches to understanding community and its relationship to and with sport. Secondly, by inviting contributions from a range of international contexts, we aimed to provide rich discussions about different sport(s) and community(ies). Thirdly, we encouraged both studies that illustrate the sport-community connection, as well as those that critique assumptions about this relationship. Cumulatively, through this approach, we aimed to provide a critical, contextual, and rich exploration of the sport-community relationship in order to stimulate future discussions across disciplines.

As the editorial team, we were thrilled with the contributions submitted to this special topic. In total, eight research teams participated. These contributions included conceptual work examining how community has been invoked and theorized (Rich et al.) as well as practical applications of methods for understanding community contexts in sport for development and peace projects (Gadais et al.). Contributions problematized participation contexts and challenged readers to consider how leisurely engagement such as neighborhood walking (Glover et al.) and recreation programs offered through University-based partnerships (Ali et al.) have implications for our understandings of community. Contributors also examined diverse contexts by interrogating the ideological underpinnings of national sport policies in Sweden (Bjärsholm and Norberg) and the features (i.e., scope and location) of private community sport organizations in London, Ontario, Canada (Doherty et al.). Publications in this collection also challenge us to think critically about the structures that frame our understandings of community. For example, authors examined mentorship for Black women coaches in community sport (Joseph and McKenzie) as well as youth development initiatives in Latino-based community sport (Robledo et al.).

Through these contributions, much of what we sought out to do was achieved. Contributors have engaged a range of theoretical perspectives from critical sociology, political economy, management, and leisure studies. Our collection is interdisciplinary and boundary-spanning in many ways. Authors also provided rich and critical analyses of the sport-community relationship. This is evident through the variety of conceptual framings (from social ecological systems and positive youth development to racial inequality) as well as the diversity of methodological approaches involved (from GIS mapping to participatory research methods). In this way, contributors have confirmed some established ways of examining sport and community, but also challenged some assumptions and conceptual applications of previous work. This collection illustrates the value of interdisciplinary work in navigating complex and messy conceptual domains.

One area where this collection does come up short is the representation and diversity of research contexts and authorship. The majority of our contributors are situated in North American Institutions, with some European and Australian representation. This aligns with previous review work that identified a similar trends in other fields of sport scholarship (11–13). This collection therefore, does represent a more narrow perspective than we initially hoped, and should be considered as such. Future work should address this limitation and continue to contribute to diverse theorizations of community and how sport is framed by political, social, and cultural contexts.

We hope that the collection of articles offered here lays the conceptual and methodological foundation for a critical and interdisciplinary body of literature that continues to challenge the ways we think about sport, community, and the possibility of a common good.

## Author contributions

All authors listed have made a substantial, direct, and intellectual contribution to the work and approved it for publication.

## Conflict of interest

The authors declare that the research was conducted in the absence of any commercial or financial relationships that could be construed as a potential conflict of interest.

## Publisher's note

All claims expressed in this article are solely those of the authors and do not necessarily represent those of their affiliated organizations, or those of the publisher, the editors and the reviewers. Any product that may be evaluated in this article, or claim that may be made by its manufacturer, is not guaranteed or endorsed by the publisher.
